# Cycling and walking for transport: Estimating net health effects from comparison of different transport mode users' self-reported physical activity

**DOI:** 10.1186/2191-1991-1-3

**Published:** 2011-07-20

**Authors:** Knut Veisten, Stefan Flügel, Farideh Ramjerdi, Harald Minken

**Affiliations:** 1 Institute of Transport Economics (TØI), Gaustadalleen 21, NO-0349 Oslo, Norway

## Abstract

**Background:**

There is comprehensive evidence of the positive health effects of physical activity, and transport authorities can enable this by developing infrastructure for cycling and walking. In particular, cycling to work or to school can be a relatively high intensity activity that by itself might suffice for maximum health gain. In this paper, we present estimates of net health effects that can be assumed for demand responses to infrastructure development. The estimation was based on comparing current cyclists/pedestrians against potential cyclists/pedestrians, applying the *international physical activity questionnaire*, which is a survey-based method for estimating *metabolic equivalent task *levels from self-reported types of physical activity, and their frequency, duration and level of intensity (moderate or vigorous).. By comparing between shares of individuals with medium or high intensity levels, within the segments of current cyclists/pedestrians and potential cyclists/pedestrians, we estimate the possible net health effects of potential new users of improved cycling/walking infrastructure. For an underpinning of the estimates, we also include the respondents' assessments of the extent to which cycling/walking for transport replaces other physical activity, and we carry out a regression of cycling/walking activity levels on individual characteristics and cycle/walk facility features.

**Results:**

The estimated share of new regular cyclists obtaining net health gains was ca. 30%, while for new regular pedestrians this was only ca. 15%. These estimates are based on the assumption that the new users of improved cycle/walk facilities are best represented by self-declared potential users of such improved facilities. For potential cyclists/pedestrians, exercise was stated as the main motivation for physical active transport, but among current regular cyclists "fast and flexible" was just as important as exercising. Measured intensity levels from physically active transport increased with separate cycling/walking facilities, and were higher for those with higher education and living in urban areas, while they were lower for those with higher BMI and higher age.

**Conclusions:**

Since the share obtaining net health gains might have a huge impact on cost-benefit analysis of new or improved infrastructure for cyclists/pedestrians, it is of importance to estimate this share. A main limitation of our estimation is the cross-sectional design. There is a need for more case studies combining surveys and objective measurement of physical activity changes, preferably before and after the construction of new infrastructure.

## Background

There is now strong evidence of the positive health effects of physical activity. Daily moderate or vigorous activity of approximately 30 minutes' duration contributes to reduced mortality and possibly to avoiding or delaying potential outbreaks of cardiovascular disease, stroke, colon cancer, breast cancer or type II diabetes [1-3]. In a large study from Copenhagen based on self-reported physical activity, medical checks and follow-up registration of fatalities, an all-cause relative mortality risk of ca. 0.72 was calculated for those cycling for transport compared to those not cycling [4].

Health effects may constitute a considerable benefit element in economic assessment of policy measures promoting cycling (and walking) for transport [3,5,6], and transport authorities can contribute to this by developing infrastructure in quantity as well as quality. Cycling to work or school is a relatively high intensity activity that by itself might suffice for maximum health gain. For some people, cycling/walking transport facilities would be a much needed arena for physical activity, i.e. for exercise that would not be taken if the transport infrastructure was inadequate [7,8].

In the first cost-benefit analysis of a new cycling/walking track network taking into account the positive health effects of physical activity it was assumed that the share of new cyclists and pedestrians obtaining net health effects would be 50% [6]. Recent WHO-based guidance on cost-benefit analysis of health effects proposes the use of the all-cause relative mortality risk estimate of 0.72, from the Copenhagen study, for those cycling for transport, and this is attributed to all new cyclists [3]. However, even though the current regular cyclists have a lower mortality risk, we lack an empirical basis for assessing the health gain for new cyclists. We do not know, a priori, whether new cyclists (or new pedestrians) just replace other types of physical activity, or whether they increase their physical activity, obtaining net positive health effects. Objective health measurement of the affected population before/after facility construction/enhancement is infeasible for most purposes.

For new cyclists/pedestrians, the potential health gains from increased cycling/walking for transport rest on two underlying assumptions: (i) that they do not already have a sufficiently high physical activity level; and (ii) that they do not just substitute cycling/walking for other physical activity. Net health gains can be expected for the share of respondents for whom these assumptions are met [1-4]. In the WHO guidance it is recommended though "that activity substitution is accounted for in economic analyses as far as possible. This means not making an assumption that any increase in cycling or walking automatically leads to an increase in total physical activity (as people may cycle more and do less of another activity as a result)" [[3], p. 9]. In the Copenhagen study the relative all-cause mortality risk for regular cyclists of 0.72 compared to non-cyclists was based on controlling for various individual characteristics, including other types of physical activity. However, some factors might have been omitted. Furthermore, it is not obvious that the potential cyclists are a representative sample of all non-cyclists, nor that the new cyclists will constitute a representative sample of all regular cyclists. Those who currently cycle for transport, in Denmark as well as in Norway, may constitute the most physically active segment of the population; it is possible that most of them would have been active even if not cycling. Potential cyclists may also constitute a relatively active segment; many of them may have other physical activity that they partially replace by cycling for transport if facilities are improved. The WHO experts proposing guidelines for economic analyses of measures increasing transportational cycling/walking, did stress that such analyses should "incorporating a factor into the calculations to allow for the possibility that the level of cycling or walking being assessed will not have increased total physical activity among some of the observed participants" [[3], p. 9].

Our paper presents a way of estimating the share obtaining net positive health effects based on questions from the so-called *international physical activity questionnaire *(IPAQ) - a survey-based method for estimating *metabolic equivalent task *(MET) levels from self-reported activity types, frequency, duration and (moderate or vigorous) intensity level [9]. Our study enables differentiation between current regular cyclists, or regular pedestrians, and potential cyclists/pedestrians, i.e. those who state that they might cycle/walk if conditions were improved. By comparing the share of individuals with medium or high intensity levels among current versus potential cyclists/pedestrians, we estimate the possible net health effects on the potential users of new/improved cycling/walking infrastructure. Thus, our estimates of the share of new cyclists/pedestrians obtaining net health effects are based on the assumption that the new users of improved cycle/walk facilities are best represented by self-declared potential users of such improved facilities. Although this approach is in no way the panacea for estimating net health gains, our contribution is a step towards increasing our knowledge of the impacts of promoting cycling and walking for transport. We include current and potential cyclist/pedestrian assessments of the extent to which cycling/walking for transport replaces other physical activity, and we carry out a regression of cycling/walking activity levels on individual characteristics and cycle/walk facility features.

## Methods

### Estimating the health effect from increased cycling/walking

In a survey-based data collection, we can include questions about current physical activity for transport as well as about all other types of physical activity. An IPAQ available at the webpage of the Karolinska Institutet in Stockholm http://www.ipaq.ki.se/ipaq.htm is a standard survey-based instrument that can be used to obtain internationally comparable data on health-related physical activity [9,10]. It comprises a set of four questionnaires, a long and a short version adaptable for either a telephone interview or self-administer (postal) format. We adapted the self-administer format to an Internet-based survey, combining the short version with active transport questions from the long version. The IPAQ applied therefore contains questions about frequency and duration of *vigorous *physical activity, *moderate *physical activity, as well as about cycling and walking for transport.

Regarding physical activity in transport, the following self-reported data are obtained:

• *x *trips of cycling per week

• *y *trips of walking per week

• *ψ *minutes of cycling per trip

• *φ *minutes of walking per trip^a^

These data are entered into a score formula for calculating the *Metabolic Equivalent Task *(MET) from cycling/walking for transport during the week. 1 MET is the metabolic equivalent task at rest (seated), for the average adult, corresponding to approximately 3.5 ml O_2_/kg body weight per minute [11]. Cycling for transport is considered a vigorously physical activity, with 6 MET per minute. Walking for transport is considered closer to moderate physical activity (3 MET), with 3.3 MET per minute http://www.ipaq.ki.se/scoring.pdf. The following scores from physical activity for transport can be obtained:

◦ Cycling MET minutes per week = 6 × minutes × trips = 6 × *ψ *× *y*

◦ Walking MET minutes per week = 3.3 × minutes × trips = 3.3 × *φ *× *x*

◦ SUM transport MET minutes per week = 6 × *ψ *× *y *+ 3.3 × *φ *× *x*

Regarding all other physical activity, the IPAQ differentiates between *vigorous *and *moderate*, and the following self-reported data are obtained:

• *s *times (days) of *vigorous *physical activities per week

• *t *times (days) of *moderate *physical activities per week

• *ζ *minutes of *vigorous *physical activity per activity carried out

• *η *minutes of *moderate *physical activity per activity carried out

Examples of vigorous physical activity are heavy lifting, heavy manual work/construction work, aerobics, fast bicycling/running, while moderate physical activities are light manual work/construction work, swimming and fast walking. Clearly, the examples were intended to give the respondent some indication, and they may vary considerably between subjects in terms of MET minutes. It is also stated that "vigorous physical activities refer to activities that take hard physical effort and make you breathe much harder than normal"; and that "moderate activities refer to activities that take moderate physical effort and make you breathe somewhat harder than normal" http://www.ipaq.ki.se/ipaq.htm. The following scores from all physical activity can be obtained:

◦ Vigorous MET minutes per week = 8 × minutes × activity = 8 × *ψ *× *y*

◦ Moderate MET minutes per week = 4 × minutes × activity = 4 × *φ *× *x*

◦ SUM all physical activity MET minutes per week = 8 × *ψ *× *y *+ 4 × *φ *× *x*

The contribution from active transport as a share of all physical activity can also be calculated.

With no possibility of following cohorts or making before-after comparisons, we opted for a comparison of transport segments within our cross-sectional setting, i.e. physical activity levels of regular cyclists/pedestrians compared to those of potential cyclists/pedestrians. Based on estimation of MET minutes per week and frequency of (vigorous) physical activity, we can classify respondents into three activity classes http://www.ipaq.ki.se/scoring.pdf[9]:^b^

◦ High (*h*) level of physical activity with two criteria for classification: a) vigorous intensity activity on at least three occasions achieving a minimum total physical activity of at least 1500 MET minutes/week; or b) seven or more occasions of any combination of walking, moderate intensity or vigorous intensity activities (including cycling) achieving a minimum total physical activity of at least 3000 MET minutes/week.

◦ Moderate (*m*) level of physical activity, with three criteria for classification: a) three or more occasions of vigorous intensity activity of at least 20 minutes per occasion; or b) five or more occasions of moderate intensity activity and/or walking of at least 30 minutes per occasion; or c) five or more occasions of any combination of walking, moderate intensity or vigorous intensity activities achieving a minimum total physical activity of at least 600 MET minutes/week.

◦ Low (*l*) level of physical activity for individuals who do not classify for either of the other two activity classes.

Reaching the moderate intensity level is considered the most important threshold for obtaining positive health effects [9,10,12,13]. However, physical activity beyond this level (reaching the high intensity level) may have additional effects [4].

### Physical activity substitution and a model of cycling/walking activity levels

It is not necessarily the case that increased cycling/walking for transport will yield net health gains, since this depends on the existing physical activity levels of potential cyclists/pedestrians [3]. Without the possibility of following a population over time in a cross-section analysis, both actual cyclists/pedestrians and potential cyclists/pedestrians can be asked to assess the extent to which physically active transport substitutes other physical activity.

Our understanding of physically active transport in cross-section analysis can be enhanced by modelling cycling/walking activity levels. In presenting regression models of walking as well as vigorous and moderate physical activity, in a Belgian sample, it was found significant effects of environmental variables, e.g. availability of pavements/paths, and individual characteristics, e.g. age [14]. In a similar regression analysis based on a British sample, it was also found such a mix of individual and environmental characteristics explaining physical activity levels [15].

### Survey development

Development of the survey was initiated in 2008 and a comprehensive test of the application of the IPAQ questions adapted to a two-wave Internet study was carried out during the summer of 2009 via e-mail recruiting from the national Internet panel of *Synovate Norway *http://www.synovate.com/about/where/europe/norway.html. In Wave 1, members of the panel answered questions about current transport, including cycling and walking; first about cycling or walking frequency during the previous year and then about the specific IPAQ for transport (from the long IPAQ version), the frequency of trips of more than 10 min during the previous week and their average duration. In Wave 2, after questions about road safety, they answered the IPAQ short version about vigorous and moderate physical activities during the previous week and their frequency and average duration. (The questions about walking and sitting were not included.)

Based on comprehensive testing in the summer of 2009, we changed the introduction compared to the IPAQ (short version). Since the Internet mode does not enable viewing questions ahead, we found it necessary to state in the introduction to the physical activity questions in Wave 2 that questions would be asked about both vigorous and moderate activity. Furthermore, a question introduced about any vigorous or moderate physical activity during the previous week, such that those stating no activity would skip the IPAQ entirely, and those indicating, e.g. only moderate physical activity, would skip the question about frequency and duration of vigorous activities. We believe these changes have improved the IPAQ's applicability to Internet-based surveys.

### The main two-wave Internet-based survey

**Figure 1:**
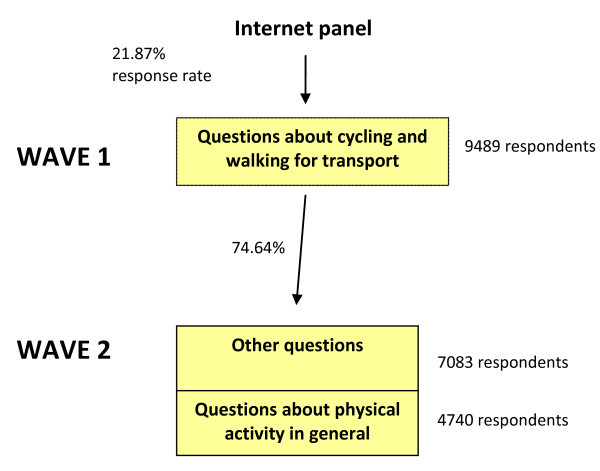
**Two-wave Internet-based survey**.

Our main survey was applied to a fairly large sample of the Norwegian population and carried out in two waves during late April and the beginning of May 2010. In Wave 1, the respondents described a recent trip they had taken for some particular transport purpose, i.e. cycling, walking or another transport mode, as well as answered other common questions about frequency and extent of cycling/walking for transport. 7082 respondents from Wave 1 also responded to Wave 2 questions about all types of vigorous and moderate physical activity choice; 21.87% of those recruited to Wave 1 responded, and the effective response rate for Wave 2 was 16.32% (Figure 1).^c^

In Wave 2, 4740 of the 7083 respondents answered questions about vigorous and moderate physical activity. The (7083-4740=) 2343 were not excluded at random; they all reported a car trip in Wave 1, and received a different version of Wave 2. But, 40% of those reporting a car trip in Wave 1 received questions about physical activity in Wave 2, together with all those who reported trips with other transport mode than car. The transport segments considered are displayed in Table 1.

**Table 1:** Transport segments, shares in Wave 2 based on reporting of cycling/walking in Wave 1

	N - wave 2	N - wave 2, receiving and responding to questions about physical activity
1a. Regularly cycling for transport (>3 times per week, during cycling season)	743 (10.5%)	731 (15.5%)
1b. Regularly walking for transport (>3 times per week), and not already in 1a	1558 (22.1%)	1216 (25.8%)
2. Irregularly cycling/walking for transport (from once a year until 3 times per week)	2956 (41.9%)	1911 (40.5%)
3. Not cycling/walking for transport, but could potentially cycle/walk given improved facilities	1253 (17.8%)	625 (13.2%)
4. Not cycling/walking for transport, and would not do it in any case	546 (7.7%)	238 (5.0%)
Total	7056	4721

For 27 (of the 7083) respondents in Wave 2 there were missing values; and for 19 (of the 4740) responding to questions about (vigorous and moderate) physical activity in Wave 2, there are missing values.

Comparing the numbers responding to, respectively, Wave 2 and the particular questions about moderate and vigorous physical activity in Wave 2, we can see that the difference is greatest for those not cycling/walking in transport. Those not responding to the question about physical activity, in Wave 2, reported a recent car trip in Wave 1. Determining "regular cycling" was based on an assessment of cycling frequency during the cycling season (approximately from April to October), since most cyclists in Norway quarantine their bicycle during winter.

The main comparison between segments will be that of the share of respondents with high (*h*) and moderate (*m*) activity levels between segment 1a of regular cyclists (or 1b of regular pedestrians) and segment 3 of potential cyclists/pedestrians [9]:

and:

We included direct questions, in Wave 1, about the extent to which cycling/walking for transport may replace other physical activity, asking both an *ex post *assessment of cyclists/pedestrians and an *ex ante *assessment of potential cyclists/pedestrians.

Finally, in Wave 1, some of the cyclists/pedestrians reported a recent biking/walking trip: the time it took, the share of the trip time on separate cycling/walking facilities, and the number of intersections (with motorized traffic). We included these two variables, together with individual characteristics, in the regression model of MET minutes per week [14,15].

## Results

### Basic statistics about the sample

In our sample of 4721 respondents, the average age was 46.3 years (from 17 to 87), with the median close to the average: 58% were men and 29% had a university degree at master's level, while another 37% had a lower university degree. Average monthly personal net income was approximately NOK 23,000 (*n *= 4460), based on taking midpoints from income intervals and setting the maximum to NOK 55,000. The median lay in the interval NOK 15,000 to 20,000.

Average age is lower for those cycling in transport, and men are slightly overrepresented. Average monthly personal net income is close to the average for all segments, but there is a significant difference between the segments in regard to education. Respondents who regularly cycle (or walk) in traffic (trips longer than 10 min) are more likely to have a university degree. Those who do not consider cycling/walking as an option have a particularly high relative share of compulsory education as their highest degree.

The average weight and BMI is lowest for regular cyclists, followed by regular pedestrians and irregular cyclists/pedestrians; however, the segment of regular pedestrians has females making up the highest share. Regular cyclists also evaluate their own health as better, compared to the others. The comparisons between segments in terms of health indicators seem consistent and intuitive.

### Physical activity levels for transport and in general

In the IPAQ it is asked about the number of times during the week the respondents carried out physical activity exceeding 10 minutes' duration. Regular cyclists indicated the highest frequency and total duration of all types of vigorous and moderate physical activity, followed by regular pedestrians. From the stated number of times physical activity of different intensities was carried out and durations of the activities, the MET can be calculated. As expected from registered activity frequency and duration, the highest average level of MET minutes per week from all types of vigorous and moderate physical activity is obtained for regular cyclists. Then follow the regular pedestrians before the irregular cyclists/pedestrians. Those stating that they would potentially cycle/walk for transport given improved facilities didn't obtain higher MET levels than those stating no interest in cycling/walking for transport.

The correlation between MET cycling/walking and MET physical activity (in total) is relatively low, i.e. only 0.41 (Pearson correlation), but is significant at the 0.05 level (2-tailed). We stress that MET cycling/walking and MET physical activity were calculated for two different weeks.

### Estimating the share obtaining net positive health effects from increased cycling/walking for transport

Based on the estimated MET minutes per week and frequency and (total) duration of various types of physical activity, we can now classify respondents in regard to intensity levels of physical activity. The classification is differentiated depending on transport segment and is displayed in Table 2. The highest shares of high and medium intensity levels of physical activity are obtained for regular cyclists - a majority from cycling for transport. An inconsistency is that the share of low intensive regular cyclists is higher for all vigorous/moderate activity than it is for physical active transport. Among regular pedestrians and irregular cyclists/pedestrians, very few qualify for high intensive physically active transport.

**Table 2:** Shares of high (*h*), moderate (*m*) and low (*l*) intensity level of physical activity carried out during the week (N = 4721)

	Intensity level from physical activity transport			Intensity level from all physical activity		
	
	*h*	*m*	*l*	*h*	*m*	*l*
1a. Regularly cycling for transport (>3 times per week, in season)	22.3%	66.3%	11.4%	32.7%	34.3%	33.0%
1b. Regularly walking for transport (>3 times per week), and not already in 1a	0.7%	43.7%	55.5%	21.2%	32.5%	46.3%
2. Irregularly cycling/walking for transport (from once a year to 3 times per week)	0.4%	3.5%	96.1%	19.2%	23.4%	57.4%
3. Not cycling/walking for transport, but could potentially cycle/walk given improved facilities				11.4%	25.9%	62.7%
4. Not cycling/walking for transport, and would not do so in any case				14.3%	23.5%	62.2%
All sample				20.3%	27.8%	51.7%

We estimate the share obtaining a positive health effect from a change to cycling or walking for transport by comparing these segments and the potential cyclists/pedestrians [9]:

and:

The indication is that the potential health gain is considerably greater for new cyclists than for new pedestrians. However, there are physically active individuals among those not cycling or walking in transport, and for some the change to cycling (or walking) for commuting or doing errands might replace other physical activity.

### Self-assessment of the extent to which cycling/walking for transport substitutes other physical activity

Among potential cyclists/pedestrians there is a larger share assessing that cycling/walking in transport would imply more time-use on physical activity, compared to the shares among those currently cycling/walking, respectively 45.3 percent vs. 28.7 percent. This might indicate different (assumed or actual) physical activity levels without cycling/walking and also that the *ex ante *perspective brings in hypothetical overstatement.

Although there is some sort of dynamic in this self-assessment combining *ex ante *and *ex post *perspectives, it may not provide any better estimates of net health gain than the cross-section comparison of MET. However, the share indicating more time-use for physical activity after commencing cycling/walking for transport could represent an alternative estimate of the share obtaining net health gain (given that they were not sufficiently physically active at the outset). The share indicating less time-use for physical activity after commencing cycling/walking for transport could indicate more efficient time-use for physical activity, respectively 18 percent among potential cyclists and 22.5 percent among regular cyclists/pedestrians.

Regarding causes stated for cycling/walking in transport, the largest share ticked exercise as the most important reason for their choosing cycling/walking for transport. However, while this share was more than a half for irregular cyclists/pedestrians, it was just above one-third for regular cyclists. For regular cyclists, a similar share ticked fast and flexible as main reasons.

### Regression of cycling/walking activity level on individual characteristics and cycling/walking facility elements

Table 3 gives the regression models (OLS) of MET minutes per week for cycling and walking, respectively, including individual characteristics and environmental/infrastructural features. The coefficient values indicate marginal effects on MET (log-transformed) from cycling or walking. Individual characteristics, as well as environmental/infrastructural features, significantly covariate with measured MET minutes per week. BMI covariates negatively with MET minutes for both cycling and walking, while university education level covariates positively. For MET from walking for transport, income, male gender, age and having children in the household covariate negatively. The latter two variables also covariate negatively with MET from cycling, but only in the model without infrastructural characteristics (model *i*). For MET from cycling, introducing the infrastructural characteristics reduces the significance of individual characteristics. The significant positive sign for residence in city compared to rural area (for cycling MET also semi-urban area) is most likely due to more facilities for cycling and walking in urban areas.

**Table 3:** Ln MET minutes per week, cycling and walking, by independent variables, OLS regression analysis

Model	ln(MET-cycling), N = 1575				ln(MET-walking), N = 4740			
	
	(*i*)		(*ii*)		(*i*)		(*ii*)	
	
	value	t-statistic	value	t-statistic	value	t-statistic	value	t-statistic
Constant	10.440	7.731	8.778	6.578	12.829	13.011	12.006	12.140
ln_age	-.497	-2.698	-.157	-.845	-1.156	-8.624	-1.001	-7.419
Male	.008	.071	.001	.010	-.250	-2.870	-.247	-2.850
University education	.574	4.560	.446	3.588	.525	5.707	.446	4.857
Children in household	-.178	-1.554	-.072	-.641	-.640	-7.020	-.624	-6.832
log_income	.073	.634	-.051	-.447	-.285	-3.295	-.321	-3.731
ln_BMI	-1.349	-4.100	-1.246	-3.869	-.802	-3.526	-.726	-3.208
Reference trip, cycle							.359	2.071
Residence in semi-urban area			.545	3.043			.088	.691
Residence in city			1.074	6.290			.400	3.388
ln_share_separated			.153	4.515			-.070	-1.579
ln_crossings_km			.185	1.781			.674	5.656
Adj. R^2^	.035		.082		.060		.076	

Rural area is reference category to semi-urban and city residency. "Reference trip cycle" indicates that the respondent reported a recent cycling trip in Wave 1 of the survey; and the share of separated cycling/walking paths and the number of crossings were based on the reported cycling or walking trip.

The shares of separate cycling/walking facilities and number of intersections from a reported actual trip (cycling or walking) were registered. The coefficient for the share of separated facilities appears with significantly positive sign only in the model for cycling MET. The coefficient of crossings per km appears with significantly positive sign, and the co-variation is particularly strong for MET walking. For the modelling of MET minutes per week walking, cycling as transport mode in the reference trip was also controlled and the coefficient has significantly positive sign.

In the regression modelling of MET minutes per week cycling or walking for transport, several characteristics of the individual and of the infrastructural features in his/her surroundings appeared with expected coefficient signs. However, a positive sign for the number of intersections per km was not as expected, although this supposed barrier was less positive for cycling. The specification of the infrastructural features was possibly too coarse, such that the intersection variable contained omitted specification of cycling/walking facility supply that was not contained in the dummy variables for the degree of urbanization.

## Discussion

Our study presents a new approach assessing cycling/walking in transport and estimating potential health gains. Surveying in a transport context enabled comparison between current cyclists/pedestrians and potential cyclists/pedestrians based on self-reported activity levels. There is very probably self-selection in transport mode choice, such that physically active people to a larger extent, ceteris paribus, choose physical active transport modes. Our study indicates that those who initiate or increase cycling/walking for transport will substitute for other physical activity a combination of saving time and increasing overall time spent on physical activity.

It is not obvious that potential cyclists who start cycling for transport will reach the average total physical activity level of existing regular cyclists. This represents additional information compared to, for example, comparison of all-cause relative mortality risk between cyclists and all others [4]. However, we certainly do not claim superior estimates. There are obvious weaknesses in our cross-section data with self-reported activity levels. The registration of physical activity in general was done approximately one week after the registration of physical active transport. Changes in the weather and a short May Day holiday for part of the sample, between the two weeks of registration in the two-wave survey, could have exacerbated differences in physical activity levels. The correlation between these two measures was approximately 0.4.

In general, people tend to underreport moderate physical activity [16,17]. Furthermore, in our case, some individuals might have omitted physical active transport when asked about physical activity in general. The underestimation of overall physical activity is also indicated from a comparison against Norwegian estimates in former studies [18]. While this error leads to a downward bias of net health gains, the effect of the underreporting of moderate activity is not so clear in our case. Regarding self-assessment of potential cycling/walking, including distance measures for work/school and shopping could possibly have been used as a type of control.

## Conclusions

We have presented a method for estimating the share obtaining net positive health effects from physically active transport based on questions from the IPAQ [9,12]. We differentiated between current regular cyclists and potential cyclists/pedestrians, and compared between the shares of individuals with medium or high intensity levels. The estimated share of new regular cyclists obtaining net health gains was ca. 30%, while for new regular pedestrians this was only approximately 15%. A lower average intensity level for walking than for cycling might partially explain this difference. Our estimates are based on the assumption that the new (and unknown) users of improved cycle/walk facilities are best represented by self-declared potential users of such improved facilities.

Regarding assessment of total time spent on physical activity when commencing physically active transport, a slightly larger share stated more time spent than less time spent, and the difference was prominent among potential cyclists/pedestrians. For potential cyclists/pedestrians, exercise was stated as the main motivation for physical active transport, but among current regular cyclists "fast and flexible" was just as important as exercising in their choosing cycling as a transport mode. This can be taken as yielding some support to the findings from the IPAQ-based comparison, that the majority of the active cyclists have substituted cycling for other exercise. Measured intensity levels from physically active transport increased with separate cycling/walking facilities, and were higher for those with higher education and living in urban areas, while they were lower for those with higher BMI and higher age. The correlation with demographic factors was consistent with results from former studies [19,20]. Thus, new/improved facilities are important for stimulating physically active transport, but there is seemingly self-selection of relatively young and fit to cycling in transport.

We believe that our contribution is a step towards increasing our knowledge of the impacts of promoting cycling and walking for transport. However, there is clearly scope for improving our application of the IPAQ questions. Self-reported physical activity combined with medical checks and follow-up registration of fatalities [4], with our differentiation between current regular and potential cyclists/pedestrians, would be promising development. Finally, the follow-up should include some measurement of physical activity changes, preferably related to infrastructure measures that could affect cycling/walking in transport. There is a need for more case studies combining surveys and objective measurement of physical activity changes, preferably carried out before and after the construction of new infrastructure.

## Abbreviations

BMI: Body Mass Index; IPAQ: International Physical Activity Questionnaire; MET: Metabolic Equivalent Task; WHO: World Health Organization.

## Competing interests

The authors declare that they have no competing interests.

## Authors' contributions

KV has made substantial contribution to conception and design, acquisition of data, interpretation of data, and has leaded the drafting of the manuscript. SF has made substantial contribution to conception and design, acquisition of data, has leaded the analysis and interpretation of data, and has been involved in drafting of the manuscript. FR has made substantial contribution to conception and design, acquisition of data, and has revised the manuscript critically. HM has made substantial contribution to conception and design, as well as to the interpretation of data, and has critically revised the manuscript. All authors have given final approval of the version to be published.

## Endnotes

^a ^"Per day" is applied in the original format http://www.ipaq.ki.se/ipaq.htm. We made changes for the Internet-based adaptation of the self-administered format of the short questionnaire version plus cycling/walking for transport. The questions about physical activity duration were posed as *per activity *(or *per trip*) rather than per day, as applied in the original version, since a pilot survey indicated misunderstanding of frequency and duration (of different activities) *per day*. Furthermore, we added an introduction clarifying that respondents would be asked about both *vigorous *and *moderate *physical activity.

^b ^In the Copenhagen study, leisure time physical activity was assessed by responses to the following statements: "(1) You are almost entirely sedentary or perform light physical activity less than 2 hours per week, ie, reading, TV, cinema; (2) You perform light physical activity 2-4 hours per week, ie, walking, cycling, light gardening; (3) You perform light physical activity more than 4 hours per week or more vigorous activity 2-4 hours per week, ie, brisk walking, fast cycling, heavy gardening, sports where you get sweaty or exhausted; (4) You perform highly vigorous physical activity more than 4 hours per week or regular exercise or competitive sports several times per week" [4].

^c ^According to Synovate Norway http://www.synovate.no, our response rate is common for their Internet panel, and they apply techniques for adjusting the sample to population figures, i.e. distributions of gender, age and regional belonging. Synovate Norway, formerly *MMI (Markeds- og Mediainstituttet) AS*, is part of the international opinion research company Synovate http://www.synovate.com.
